# Closing the Loop on Personal Protective Equipment: Collection, Polymer Recovery, and Circular Pathways for Post-Consumer PPE

**DOI:** 10.3390/polym18030336

**Published:** 2026-01-27

**Authors:** Giulia Infurna, Marinella Levi, Loredana Incarnato, Nadka Tz. Dintcheva

**Affiliations:** 1 Department of Engineering, University of Palermo, Viale delle Scienze, Ed. 6, 90128 Palermo, Italy; 2 Department of Chemistry, Materials, and Chemical Engineering “Giulio Natta”, Politecnico di Milano, 20133 Milano, Italy; marinella.levi@polimi.it; 3 Department of Industrial Engineering, University of Salerno, Via Giovanni Paolo II 132, I-84084 Fisciano, Italy; lincarnato@unisa.it

**Keywords:** personal protective equipment (PPE), decontamination, mechanical recycling, chemical recycling, elastomer recovery

## Abstract

The rapid growth of personal protective equipment (PPE) consumption has generated unprecedented volumes of polymer-based waste, posing a major challenge to the transition from a linear to a circular economic model. The challenges associated with PPE recycling are strongly linked to the sector of origin—including healthcare, laboratories, cleanrooms, and food processing—as this factor determines contamination levels and critically influences subsequent recycling steps. PPE waste originating from the healthcare sector requires stringent decontamination processes, which directly affect the final properties of recycled materials and their suitability for upcycling or downcycling applications. Another decisive factor is source segregation, together with labeling and sorting, given the intrinsic material heterogeneity of PPE, which commonly includes polypropylene (PP) masks, polycarbonate (PC) protective eyewear, and nitrile butadiene rubber (NBR) gloves. Mechanical and chemical recycling routes, including processes specifically developed for elastomeric materials, play a complementary role depending on the cleanliness and composition of the waste streams. The potential for downcycling and upcycling of recycled PPE is closely linked to polymer integrity and process compatibility. When appropriate segregation strategies and tailored recycling technologies are implemented, PPE waste can be effectively diverted from incineration. Under these conditions, PPE—once emblematic of single-use culture—can become a representative example of how complex polymer products may be reintegrated into sustainable material loops, contributing to resource efficiency and circular-economy objectives.

## 1. Introduction

The pressing need for a transition to a circular economy has been underscored by the EU’s Green Deal and Italy’s National Recovery Plan [1,2,3], with the objective being to conserve resources by reducing the demand for raw materials and energy, minimizing waste by keeping resources in use, and avoiding disposal challenges. The model under consideration is diametrically opposed to the conventional linear economic model, which functions on the basis of the “take, make, use, dispose” principle. The aforementioned model is responsible for the depletion of finite resources and waste accumulation, which in turn has a deleterious effect on the environment [4,5]. In an effort to reduce the consumption and littering of single-use items, governments around the globe have launched various initiatives, but even so, it is predicted that plastic waste will double by 2030 [6,7,8,9]. Every year, around 380 million tons of plastic are produced around the world. Polyolefins account for more than 60% of all municipal solid waste. Nevertheless, less than 20% of these plastics are reprocessed after use, while the majority are either burned or discarded in landfills and oceans, resulting in a waste of precious resources [10]. A frequently disregarded category of waste in recycling processes includes personal protective equipment (PPE). 

PPE has become integral to modern industry and healthcare, enabling safe operations in hospitals, semiconductor manufacturing, food processing, pharmaceuticals, and chemical laboratories. Although public attention peaked during the COVID-19 pandemic, global PPE consumption has remained structurally high due to increased regulatory stringency, the growth of cleanroom-dependent industries, and the widespread adoption of disposable protective items. This shift has created an enduring waste-management challenge: how to responsibly handle vast quantities of polymer-based PPE after use [11,12,13,14].

Post-consumer PPE waste differs markedly from conventional plastic waste. Many items are contaminated, i.e., biologically, chemically, or mechanically. In addition, PPE frequently employs multilayer or composite structures designed for high performance rather than recyclability, such as meltblown polypropylene (PP) filtration layers in surgical masks, nitrile butadiene rubber (NBR) in gloves [15], polyethylene (PE)–polypropylene laminates in gowns [15,16,17], and polycarbonate (PC) or polyethylene terephthalate glycol (PETG) in face shields [18,19]. These complexities have contributed to the dominance of incineration, which remains the default disposal route in many institutional settings, especially in healthcare [20]. However, recent research demonstrates that both mechanical and chemical recycling of PPE polymers are increasingly feasible, provided that collection, decontamination, and sorting protocols are carefully designed. At the same time, emerging material innovations (e.g., devulcanized NBR feedstocks [21], nanofiber recovery from meltblown layers [22], or pyrolysis-based oil production from mask waste [23]) suggest opportunities not only for downcycling but also for genuine upcycling into high-performance materials or value-added products [24,25,26,27,28]. 

**Scheme 1:**
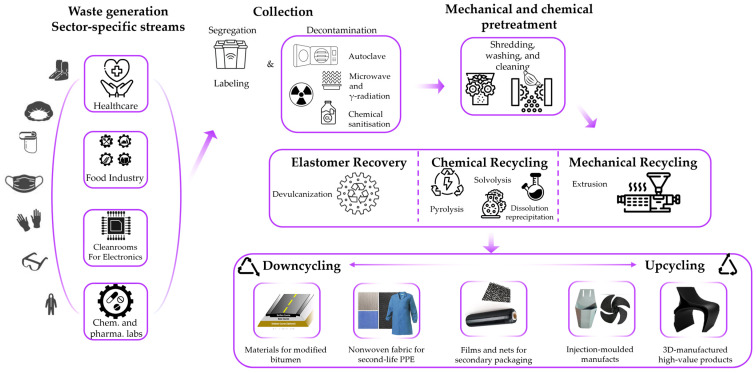
Steps in the recycling process of PPE.

As is shown in Scheme 1, this article provides a thorough overview of the current landscape of PPE waste generation, collection strategies across various sectors, post-consumer treatment pathways, available recycling technologies, and potential upcycling and downcycling solutions. Waste generation originates from healthcare, the food industry, electronic cleanrooms, and chemical and pharmaceutical laboratories. Particular attention is paid, in this overview, to how the origin and use context of PPE and polymer-based products determine the required level of sanitization or decontamination, thereby directly influencing the feasibility and selection of subsequent recycling routes. After collection, materials undergo segregation and labeling, followed by decontamination processes such as autoclaving, microwave and γ-radiation treatment, or chemical sanitization, depending on the contamination risk associated with each sector. Subsequently, mechanical and chemical pretreatments—including shredding, washing, and cleaning—prepare the materials for different recovery strategies. These include elastomer recovery through devulcanization, chemical recycling via pyrolysis, solvolysis, or dissolution–reprecipitation, and mechanical recycling through extrusion.

The combination of waste origin, sanitization requirements, and pretreatment severity ultimately governs whether materials can be directed towards mechanical or chemical recycling routes—or excluded from recycling altogether—thereby determining the extent to which downcycling or upcycling pathways can be realistically achieved.

## 2. PPE Waste Generation and Sector-Specific Streams

Personal protective equipment enters the waste stream from a wide spectrum of operational environments, each with distinct contamination profiles and regulatory constraints. Beyond the healthcare and hospital sector, traditionally the most studied source of PPE waste, significant volumes originate from chemical and analytical laboratories, pharmaceutical cleanrooms, electronics manufacturing facilities operating under ISO-class cleanroom conditions, and food-contact environments throughout both production and distribution chains. This diversity in operational contexts results in equally diverse material compositions, exposure histories, and disposal requirements, making PPE waste a non-uniform and technically complex stream to assess for potential recovery or recycling.

Hospitals stand as the primary and most voluminous global source of discarded PPE, a waste stream whose scale escalated drastically during the pandemic, fundamentally altering the economics of healthcare waste management. Projections suggest that the global PPE market size, valued at $58.64 billion in 2023, will continue growing, particularly the respiratory protective equipment segment expected to surpass $16 billion by 2032, ensuring persistently high consumption rates [29]. The core challenge lies in the complex material composition of PPE, including multilayered PP in masks and gowns, NBR or latex in gloves, and PETG/PC in face shields [30], coupled with the overriding priority of infection control [31,32]. In fact, several studies have demonstrated that used PPE may retain a significant biological load after clinical use, thereby constituting a potential risk factor for infection control within healthcare facilities [33,34,35]. Regulatory mandates, like the WHO guidelines and the EU Directive 2008/98/EC [36], classify much of this waste as biohazardous, necessitating energy-intensive and costly pretreatment processes such as autoclaving or incineration [37]; disposal fees for regulated medical waste (RMW) can be 10 times higher than landfilling, often leading to polymer degradation that renders mechanical recycling unfeasible [38,39]. Crucially, studies indicate that hospitals frequently overpay by up to 70% for waste management due to the misplacement of general trash into RMW bins, making waste segregation a critical financial and environmental imperative [40,41]. This economic pressure has spurred the development of innovative pilot programs focusing on source segregation of “non-infectious” PPE from low-risk or administrative areas, creating a clean feedstock suitable for circular pathways. Furthermore, advanced solutions like chemical recycling (pyrolysis) are emerging as a viable method to process contaminated, mixed-polymer streams (like PP [42] and NBR mixtures [43]), breaking them down into valuable hydrocarbon feedstocks for new plastics or fuels, offering a high-tech avenue to close the loop where mechanical recycling fails [44,45].

In the broader effort to reduce the environmental footprint of high-tech manufacturing, the management of cleanroom waste is emerging as a critical yet often overlooked challenge [46,47]. Electronics and semiconductor fabrication facilities depend on extremely controlled environments, and this reliance translates into large volumes of single-use PPE. Items such as Tyvek (DuPont de Nemours, Inc., Wilmington, DE, USA) suits made of high-density polyethylene (HDPE), nitrile gloves, and polypropylene or polyester masks constitute a substantial, continuous waste stream.

Although these waste streams are typically cleaner than those generated in healthcare settings, where biological contamination is the primary concern, they still present nontrivial challenges. During production, PPE can accumulate traces of solvents, ultra-fine particulate matter, photoresist residues, and metal contaminants (for example, copper, tungsten, or rare earth elements). These residues can interfere with downstream recycling processes, affecting the selection of suitable decontamination methods, the energy required to remove embedded contaminants, and ultimately the economic viability of mechanical recovery or chemical depolymerization [48].

Despite these challenges, cleanroom-specific PPE tends to be manufactured from more uniform and well-characterized polymer streams compared to the mixed plastics typical of municipal or clinical waste. This relative material homogeneity, especially the prevalence of single-polymer items such as HDPE suits and polypropylene masks, makes the sector a promising candidate for developing closed-loop or semi-closed-loop recycling pathways. In fact, the cleanroom-specific PPE is manufactured under tightly controlled material specifications. Common cleanroom garments, such as HDPE coveralls (e.g., Tyvek^®^) and single-polymer polypropylene masks, are produced as mono-material items with well-characterized compositions, a feature highlighted both in industrial documentation and in recent life cycle assessment (LCA) studies comparing reusable and disposable coveralls [49]. This material homogeneity is significant: mechanical recycling processes perform best when feedstocks consist of single polymers rather than mixed-plastic blends, which otherwise degrade material quality and limit circularity. Moreover, several assessments indicate that non-contaminated PPE made of pure HDPE or PP can be successfully reprocessed, provided dedicated collection, sorting, and decontamination pathways are in place [49]. While challenges remain, particularly contamination risks and the degradation behavior of some polymers, the relative purity of cleanroom PPE makes the sector an unusually strong candidate for piloting closed-loop or semi-closed-loop recycling systems compared to the heterogeneous waste streams typical of general healthcare or municipal contexts. Within industrial ecosystems, this opens opportunities for creating targeted take-back schemes, integrating advanced sorting technologies, and designing PPE with recyclability in mind from the outset, thereby supporting circular material flows without compromising cleanroom performance standards.

PPE used in food-processing environments, such as hair nets, gloves, aprons, sleeves, and masks, is typically employed in highly controlled contexts where contamination risks are relatively low compared to healthcare settings. Food safety regulations, such as Hazard Analysis and Critical Control Points (HACCP) and related preventive control frameworks, focus on hygiene and cross-contamination prevention [50,51]. However, food-processing PPE is not generally exposed to hazardous biological agents, pharmaceuticals, or chemically aggressive substances [50]. As a result, post-consumer PPE originating from the food sector tends to be cleaner and more compositionally uniform with respect to the healthcare and hospital sector, with a predominance of polyolefin-based materials like polyethylene and polypropylene. This cleaner waste stream is significantly more compatible with mechanical recycling pathways, both in terms of contamination levels and polymer purity.

In fact, several industrial food-processing facilities have already introduced dedicated internal collection systems to segregate used PPE by material type [52]. These closed-loop or semi-closed-loop systems ensure a consistent, traceable feedstock that can be reprocessed into recyclates with fewer downstream treatments. Such initiatives demonstrate the practical feasibility of integrating PPE waste from food environments into existing recycling infrastructures, supporting circular-economy goals without compromising safety or compliance.

Managing PPE waste in the food-processing sector is considerably easier than in other industries because the materials are generally cleaner and more uniform. Since items like hair nets, gloves, aprons, and sleeves are used in controlled environments and are not exposed to hazardous chemicals or high-risk biological agents, they do not require special disposal procedures. This means they can be handled much like regular industrial plastics rather than as dangerous waste [53].

The more orderly the collection step, the easier it becomes to transform the material into a high-quality recyclate. All of this contributes to a smoother, more sustainable waste-management strategy: disposal costs are lower, recycling becomes technically and economically feasible, and companies can reintegrate recycled plastics into new products or components. In practice, this turns what was once a linear flow of disposable materials into an opportunity for circularity, without complicating safety or compliance requirements.

Chemical laboratories, pharmaceutical manufacturing sites, and multi-purpose industrial plants generate PPE waste streams characterized by high heterogeneity in both polymer composition and contaminant profiles. Typical items include nitrile or neoprene gloves exhibiting adsorption of organic solvents or reactive intermediates; nonwoven wipes contaminated with APIs, catalysts, or analytical reagents; and polycarbonate face shields subject to mechanical abrasion, thermal deformation, or deposition of fine particulates [54,55].

The principal constraint in managing these waste outputs is the variability and incompatibility of contaminants with conventional mechanical recycling processes. Solvent-exposed elastomers may undergo swelling, embrittlement, or plasticizer migration, altering rheological behavior during melt processing. Residual pharmaceutical compounds or fine powders can cause volatile emissions, thermal degradation, or the formation of process-inhibiting char during extrusion. In several jurisdictions, the mere presence of API residues triggers hazardous-waste classification, automatically excluding the material from standard polymer-recovery pathways.

The scientific literature indicates that post-use PPE represents a complex contamination interface, where biological and chemical agents accumulate as a function of sector-specific activities, exposure pathways, and contamination-control strategies. In the healthcare sector, PPE contamination is primarily biological. Multiple studies conducted during the COVID-19 pandemic demonstrated that viral RNA was frequently detectable on used PPE. Xia et al. [56] reported that approximately 50.9% of PPE surfaces were positive for SARS-CoV-2 RNA, with contamination most prevalent on shoe covers and gloves, suggesting significant environmental deposition in addition to direct patient contact. Peng et al. [57] further observed positivity rates up to 74.7% across PPE items, with higher viral loads indicated on gloves and shoe soles. Beyond viruses, bacterial and fungal contamination of reusable protective garments is also common; Balter et al. [58] documented that more than 50% of radiation-protective garments were colonized with bacteria or fungi post-use, predominantly skin-associated microorganisms.

In food-processing environments, PPE such as gloves and aprons can become contaminated with microorganisms relevant to food safety. Studies indicate that damaged or prolonged-use gloves harbor significantly higher bacterial loads, including Bacillus spp., Staphylococcus aureus, Listeria monocytogenes, and Pseudomonas aeruginosa, with total aerobic counts reaching up to 10^3^ Colony Forming Units, CFU, per glove [59]. Moreover, outbreak-oriented assessments in ready-to-eat food facilities have documented that gloved hands can exhibit mean aerobic colony counts of approximately 4.0 × 10^4^ CFU/25 cm^2^ and maximum counts of up to 1.2 × 10^6^ CFU/25 cm^2^. These counts exceed those observed on bare hands, thereby demonstrating the potential for PPE to carry and transfer high microbial loads if hygiene practices fail [60]. 

In contrast, cleanroom environments for high-tech electronics aim to maintain near-sterile conditions, minimizing biological contamination. Smith et al. demonstrated that bacterial contamination of operator garments can occur, primarily due to human skin flora transfer during donning and handling [61]. Although microbial loads on PPE are typically near detection limits due to stringent environmental-control protocols, chemical contaminants represent a significant concern. Airborne molecular contamination (AMC), including volatile and semi-volatile organic compounds (VOCs/SVOCs), persists even at ultra-low concentrations and can affect product integrity and facility air quality [62]. Human-related VOC emissions such as acetone and isoprene have been measured in controlled cleanroom settings despite PPE use [63].

Finally, chemical laboratories represent a scenario in which PPE contamination is predominantly chemical. In these settings, microbial contamination is negligible except when biological materials are handled [64]. Laboratory PPE can accumulate a diverse array of chemicals, including organic solvents (e.g., acetone, methanol, chlorinated compounds), strong acids and bases, aromatic or halogenated organics, metal salts, and reactive intermediates [65,66].

For this reason, facilities typically implement source-segregation protocols (sorting by origin), wherein PPE is separated based on operational zones such as synthesis areas, cleanrooms, analytical labs, or packaging units. Only substreams originating from low-risk or non-reactive zones meet the criteria for potential recyclability. Even within these substreams, pretreatment steps, chemical deactivation, solvent desorption, aqueous or surfactant-based washing, or thermal conditioning are often required to reduce residual contamination below regulatory or process-tolerance thresholds.

These interventions, however, significantly impact material yield, process economics, and life cycle impacts, and frequently generate secondary effluents requiring controlled disposal. As a result, the proportion of PPE waste from laboratory and industrial environments that can be routed to recycling remains structurally low. Feasibility is generally restricted to mono-material, minimally contaminated fractions (e.g., polyolefin aprons from packaging areas), while the majority requires incineration with energy recovery or other regulated disposal methods to maintain compliance and prevent cross-contamination across the waste-management chain.

## 3. Collection and Sorting of Post-Consumer PPE

### 3.1. Segregation Strategies

High-quality recycling or safe disposal of PPE waste depends critically on collection and segregation at source. Without a robust upstream system, downstream recycling (or safe disposal) becomes impractical or unsafe. This problem is particularly relevant in environments such as hospitals/cleanrooms, food-processing plants, and chemical or pharmaceutical laboratories, where PPE waste streams may contain heterogeneous materials and risk contamination.

According to European guidance, used PPE, e.g., masks, gloves, coveralls, must be managed with appropriate segregation depending on risk [36,67]. The ISPRA (Italian Institute for Environmental Protection and Research), in its 2020 report [68], indicated that PPE waste from non-infectious/domestic use may be classed as non-hazardous (and managed as general waste), whereas PPE from contexts with infection risk or potential hazard must be handled as special/infectious waste. International guidelines for healthcare waste also prescribe segregation at source, using color-coded containers or bags, and dedicated waste streams depending on type (infectious, chemical, general plastic, sharps, etc.) to prevent cross-contamination and ensure safe downstream handling [69]. As highlighted in the healthcare waste: collection, storage, and disposal guidance, bins should be leak-proof, puncture-resistant, and clearly labeled; waste should be collected frequently and not mixed with general waste or recyclables [70]. Although the literature on PPE collection specifically in food-processing plants or cleanrooms is limited, similar principles apply, Industrial and food-processing environments often handle multiple PPE types simultaneously (nitrile gloves, protective suits, masks, face shields). Dedicated containers for each type reduce contamination risk and facilitate potential recycling.

The handling of medical waste is subject to a number of worldwide rules and recommendations that establish frameworks for secure handling, processing, and disposal methods [31,71,72,73]. The World Health Organization (WHO) has established guidelines for medical waste management, which serve as the primary international reference [71,74]. These guidelines cover a variety of topics. These include waste segregation and storage, treatment technologies, and final disposal methods. Although the WHO guidelines have obtained a high level of acceptance, they encounter difficulties in practical implementation, particularly in settings with restricted resources.

Effective recycling of post-consumer PPE requires on-site waste collection and sorting, as well as post-consumer sorting methods, especially to manage heterogeneous polymer compositions, contamination, and composite structures. Table 1 compares the main sorting technologies currently applicable to mixed PPE waste streams—namely near-infrared (NIR) spectroscopy, Raman spectroscopy, tribo-electrostatic separation, and manual sorting—based on sorting accuracy, throughput, cost, and operational suitability.

The reported performances represent typical industrial or pilot-scale ranges and are influenced by material condition, including surface contamination, color, and the presence of multilayer or composite PPE. While spectroscopic methods can achieve high identification accuracy under controlled conditions, their throughput and cost vary significantly, whereas tribo-electrostatic and manual sorting approaches offer lower accuracy but reduced complexity and capital requirements.

In the case of “uncontaminated” PPE (e.g., used in the food industry, non-clinical cleanrooms), regulations may require it to be treated as ordinary waste or common plastics, but this depends heavily on risk assessments and local authorizations. Even when separate containers are used, the lack of traceability (labeling, registration, batch tracking) makes it difficult to ensure that different types of polymers or levels of contamination are kept separate until recycling: this increases the risk of mixing and compromises the quality of recycling. In chemical or pharmaceutical contexts, laboratory waste (reagent residues, solvents, biological residues) greatly complicates management: this waste is often classified as hazardous and requires specific treatment (autoclaving, incineration, disposal as chemical waste). Relevant guidelines (e.g., for waste management in healthcare settings) state that containers for biological or chemical waste must be rigid, puncture-resistant, and, if necessary, sterilized before disposal.

### 3.2. Decontamination Requirements

The disposal of PPE inherently involves a treatment or decontamination step, regardless of whether the waste stream is destined for recycling or final disposal. Because PPE is exposed to sector-specific contaminants, each stream requires sanitization procedures tailored to its risk profile. These treatments strongly influence downstream parameters, including material quality, degradation behavior, processing costs, and the overall feasibility of mechanical or chemical recycling.

In the medical sector, treatment is essential to mitigate the hazards associated with infectious waste. Insufficient or incorrect decontamination can lead to well-documented consequences such as exposure to toxic residues, bacterial or fungal infections, atmospheric release of harmful by-products, leaching into soil and groundwater, bioaccumulation of persistent substances, and broader ecological damage [80].

In contrast, PPE used in semiconductor cleanrooms, food-processing facilities, and chemical laboratories requires different approaches. In these settings, decontamination serves a dual purpose: ensuring both worker and product safety while preserving the physical and chemical integrity of polymer-based protective materials. Unlike medical PPE, where high biological loads often necessitate aggressive sterilization, industrial PPE is typically contaminated with chemical residues, volatile process solvents, particulate matter, or food-derived acids and lipids. These contaminants necessitate alternative treatment strategies, which may include solvent wiping, dry-heat exposure, controlled-temperature thermal cycles, or mild disinfectants. Because these processes interact directly with material structure, they drive distinct degradation pathways that influence recyclability, mechanical performance, and lifetime.

Several treatment methods are currently employed to manage PPE waste, each with specific advantages and limitations. Although these approaches differ in complexity and operational requirements, appropriate treatment can substantially reduce the hazards associated with contaminated polymer-based equipment. Commonly used technologies include thermal methods such as incineration, autoclave cycles, and microwave-assisted disinfection, alongside non-thermal techniques like chemical disinfection, solvent-based cleaning, and mechanical decontamination. Although incineration is traditionally classified as a treatment/disposal method, it also functions as a form of energy recovery, converting the calorific value of polymer-based PPE into usable heat, and this will be discussed further in the next chapter. Within a recycling-oriented framework, it often remains the only viable end-of-life option for heavily contaminated or high-risk PPE streams, where the potential for chemical exposure, cross-contamination, or infection precludes safe mechanical or chemical recycling. The effectiveness and suitability of each method depend on both the nature of the contaminants—and thus on the origin stream of the waste, whether biological, chemical, or particulate—and the polymer composition of the PPE itself. Consequently, selecting an appropriate treatment strategy is crucial not only for ensuring safety but also for preserving material integrity when recovery, recycling, or further handling is required.

#### 3.2.1. Autoclave Disinfection

Autoclave disinfection relies on the combined action of heat and pressurized steam to inactivate microorganisms [80]. Although it operates at lower temperatures than more aggressive thermal methods, its effectiveness is achieved through controlled pressure and prolonged exposure. A standard cycle typically involves 60 min at 121 °C and 1 bar, followed by an additional 60 min phase at 134 °C to ensure complete disinfection. The overall performance of an autoclave depends on several interconnected factors, such as maintaining the required temperature range, ensuring adequate steam penetration into the waste, managing the load volume, defining the appropriate cycle duration, and achieving efficient air removal from the chamber [73].

Among its advantages, autoclaving is characterized by relatively low operational costs, suitability for biological testing of waste, and the generation of fewer hazardous residues compared with chemical treatments. However, there are also limitations: the physical characteristics of the treated waste generally remain unchanged, the method is not suitable for all types of waste, and the composition of air emissions during the process is not fully characterized. Autoclaving cannot be properly performed for industrial PPE made of polyolefins such as PP suits, PE aprons, and HDPE cleanroom garments. Exposure to saturated steam at 121–134 °C accelerates oxidation, chain scission, and crystallinity loss, especially in PP, which undergoes thermo-oxidative degradation at relatively low oxygen concentration [73]. Thus, while autoclaving is technically feasible, repeated cycles substantially reduce the mechanical properties required for reuse or mechanical recycling [81,82].

#### 3.2.2. Microwave Disinfection

Microwave disinfection relies on the use of low temperatures combined with high-intensity microwave radiation to inactivate microorganisms and degrade organic substances. The microwaves induce molecular bond vibrations, promoting the breakdown of contaminants while consuming relatively low amounts of energy and minimizing emissions, which makes the method more environmentally friendly than some conventional treatments. Disinfection typically occurs at temperatures between 177 and 540 °C, using electromagnetic waves with wavelengths ranging from 1 mm to 1 m and frequencies between 300 and 3000 MHz [83].

The effectiveness of microwave disinfection depends on a number of operational factors, such as the characteristics and moisture content of the waste, the strength of the microwave source, the exposure time, and the degree of mixing within the treatment chamber. Moreover, the microwave disinfection contributes to volume reduction of the treated waste and generates no liquid discharge, simplifying handling and downstream management. However, the method also presents several limitations: it involves high capital costs, may increase the weight of treated waste, is not applicable to all waste types, poses a risk of exposure to operators, and the composition of air emissions during treatment is not fully characterized. These considerations must be weighed carefully when selecting microwave disinfection as a treatment strategy for PPE or other polymer-based waste streams [73].

#### 3.2.3. Gamma Radiation

Gamma radiation is a method of sterilization that uses high-energy ionizing photons to inactivate microorganisms. Unlike thermal-based methods such as microwaving or autoclaving, gamma radiation does not rely on heat; instead, it induces direct ionization of molecules and generates free radicals, which disrupt the chemical bonds in microbial DNA and other cellular components. This makes gamma radiation highly effective against a wide range of biological contaminants, including those resistant to heat or chemical treatments. However, exposure to gamma rays can also significantly affect polymer-based PPE. Materials such as polypropylene, polyethylene, and polycarbonate may undergo chain scission, crosslinking, oxidative degradation, and embrittlement, even at ambient temperatures [84,85,86]. Consequently, while gamma radiation offers reliable sterilization, its application to industrial PPE requires careful consideration of material type and desired post-treatment properties, particularly if the equipment is intended for reuse or recycling.

#### 3.2.4. Chemical Disinfection

Chemical disinfection is a widely used method for inactivating microorganisms and controlling pathogens through the application of chemical agents. It is traditionally applied to liquid infectious waste, like blood, urine, and wastewater, but it can also be used to decontaminate polymer-based PPE in some industrial settings [87]. Common disinfectants include 1% bleach solutions or 0.5% diluted active chlorine solutions, while other agents such as lime, ozone, ammonium salts, and peracetic acid may also be used, depending on the target contaminants [72].

The effectiveness of chemical disinfection depends on several operational factors, including pH, contact time, thorough mixing of the waste with the disinfectant, and whether the process is operated in a recirculation or flow-through mode. During treatment, operators may be exposed to volatile chemicals or suffer skin and eye irritation, necessitating proper safety measures. The process generates both liquid and solid residues; liquids are typically discharged into sewer systems, while solids are disposed of in landfills [88]. The chemical disinfection is time-efficient, contributes to volume reduction, and can effectively remove odors from the waste stream. However, the method also has several limitations, including high capital costs, inapplicability to all waste types, uncertain air emissions, and the need for careful storage and handling of hazardous chemicals.

Chemical disinfection is the predominant approach outside the medical sector. Alcohol wipes, quaternary ammonium compounds, and mild oxidants are used in electronics cleanrooms and food-industry packaging lines. While these agents effectively remove residues without high heat exposure, they interact differently with polymer classes: NBR gloves and other elastomeric PPE show swelling, plasticizer migration, and reduced tensile strength after repeated alcohol exposure [89]. Conversely, polyolefins such as PP and PE exhibit high chemical resistance and are broadly compatible with solvent-based decontamination, provided exposure times are controlled.

Across industrial sectors, several global assessments of PPE recycling highlight that decontamination-induced degradation is a primary barrier to high-quality mechanical recycling [90,91]. If sterilization or cleaning alters molecular weight distribution, crystallinity, or additive content, the recycled output exhibits lower melt strength and reduced suitability for reprocessing. Available case studies provide quantitative evidence that decontamination treatments primarily modify PPE-derived polypropylene through chain scission and oxidation-driven structural reorganization. In fact, the irradiation-based sterilization of FFP2 masks results in a reduction in macromolecular chain length, accompanied by an increase in crystallinity—particularly under gamma irradiation in air, where homogeneous radio-oxidation occurs [92]. Chemical and thermal treatments (e.g., NaClO, H_2_O_2_, autoclaving) applied to PP-based gowns and wraps result in a measurable decrease in molecular weight (approximately 2–7%), while melting temperature and tensile strength are largely preserved, although crystallization kinetics are altered [37]. The use of recycled PP from face masks that have been sanitized with aqueous hypochlorous acid has been found to result in a number of changes to the material [88]. Firstly, the viscosity and molecular entanglement density are reduced. Secondly, there is a slight increase in the crystallization temperature. Finally, there is a decrease in storage modulus. Therefore, aligning the decontamination method with the specific polymer type, and the contamination profile of each industrial sector, is essential to preserving downstream recyclability.

## 4. Post-Consumer Treatment

It is important to highlight that effective recycling of PPE waste requires careful preparation of the feedstock. Pretreatment processes—whether mechanical or chemical—aim to improve the processability, purity, and final properties of the recycled material by addressing heterogeneity, contamination, and polymer integrity. Mechanical pretreatment typically involves physical operations such as shredding, washing, drying, and densification to homogenize the waste stream, whereas chemical pretreatment employs solvent-based washing and selective dissolution/precipitation to recover high-purity polymers, even from contaminated or composite materials. A comparative overview of these two approaches, including their main advantages, limitations, and applicability, is provided in Table 2.

### 4.1. Mechanical Pretreatment

Mechanical pretreatment is a fundamental step in stabilizing PPE waste streams before recycling, ensuring that heterogeneous polymeric materials can be processed effectively. Common operations include shredding to increase surface area and homogenize feedstock, washing to remove residues and sterilizing agents, drying to prevent hydrolytic degradation during melt processing (particularly relevant for PET-based face shields), and densification or agglomeration to improve flow properties for extrusion. PPE waste is highly heterogeneous, consisting of films, nonwovens, elastomeric gloves, and rigid plastics, each with different mechanical and thermal behaviors.

Evidence from polymer recycling studies demonstrates that mechanical pretreatments are not merely preparatory. Shredding and washing can introduce structural defects, alter crystallinity, and in some cases generate microplastic particles, all of which influence the quality of the recycled material [93,94]. Hard-plastic waste studies have shown that incomplete homogenization or residual contamination leads to increased melt flow variability and reduced mechanical performance of recycled blends [95]. Similarly, research on polymer-based PPE and elastomers indicates that washing or chemical exposure can induce swelling, plasticizer migration, and reductions in tensile strength, particularly for NBR gloves, while polyolefins like PP and PE show high chemical resistance and maintain integrity if exposure times are controlled [89,91].

Together, these findings highlight that pretreatment must be carefully tailored to the material type, feedstock heterogeneity, and contamination profile. Failure to achieve adequate shredding, washing, and homogenization can compromise downstream processing, resulting in recycled PPE with lower melt strength, uneven extrusion, or inconsistent mechanical properties. Given the scarcity of studies specifically on PPE, results from general plastic waste streams can be used as analogous evidence, but targeted experimental work on PPE-derived materials is necessary to optimize pretreatment protocols and ensure high-quality recyclates.

### 4.2. Chemical Pretreatment

Chemical pretreatment, involving solvent washing and selective dissolution/precipitation, is emerging as a key enabler for recycling polymer-based PPE and other post-use plastic waste, especially when streams are contaminated or composed of composite/multilayer materials. The main goal of these processes is to remove surface contaminants (oils, inks, lubricants, disinfectants), residual additives (plasticizers, pigments, fillers), and non-polymer components, thereby recovering a clean polymer suitable for reuse or re-extrusion.

In a typical workflow, waste plastics (e.g., PE, PP, HDPE) are first subjected to a solvent wash that dissolves soluble contaminants or coatings without affecting the polymer matrix. Subsequently, the polymer itself is dissolved in a suitable hot solvent; after filtration to remove insoluble impurities (other plastics, fillers, dirt), an antisolvent or a temperature/pressure swing is employed to precipitate the target polymer. This dissolution–precipitation cycle can yield high-purity resins, often with mechanical and thermal properties comparable to virgin material [96,97].

For example, a recent study demonstrated that using natural solvents (terpenes) to dissolve and then precipitate HDPE can produce recycled material with minimal alteration in crystallinity, thermal behavior, and chemical composition compared to virgin resin [98]. Similarly, experiments on coated polypropylene films from food packaging showed that selective dissolution using KOH or methanol effectively removed metallic or lacquer coatings without compromising the PP backbone, indicating that analogous approaches could work for PPE made of PP/PE [99].

The benefits of chemical pretreatment in this context are several:

1. High-purity recyclate: removal of dyes, additives, fillers, coatings, and contaminants improves the quality and applicability of the recycled polymer.
2. Preserved polymer integrity: because the process does not involve high-temperature melting or oxidative stress, the polymer chains remain chemically intact, minimizing degradation [96].
3. Suitability for mixed or contaminated waste streams: even when waste is a composite (films, coatings, multilayer materials), the selective dissolution can disentangle polymer from contaminants/additives, making recycling feasible.

However, applying chemical pretreatment to PPE waste also introduces challenges. Mixed-modality waste streams—combining films, nonwovens, elastomers, and rigid plastics—complicate solvent selection and require prior sorting to avoid cross-contamination, while additives, fillers, or other non-polymer components (e.g., metal clips, elastics, elastic bands) may not dissolve or may remain as impurities after precipitation, reducing purity and complicating downstream processing [100]. Additionally, solvent-based processes demand careful solvent recovery and reuse to ensure environmental and economic sustainability, as many traditional solvents are volatile and potentially harmful; the transition towards “green solvents” (e.g., terpenes like limonene and α-pinene) is therefore promising, having already demonstrated effective dissolution of HDPE with minimal solvent loss and low energy input [98].

Chemical pretreatment via solvent washing and selective dissolution/precipitation offers a viable pathway to recover high-quality polymers from PPE and other contaminated plastic streams, preserving polymer integrity and enabling circular reuse. Nonetheless, the approach demands stringent feedstock sorting, solvent management, and careful process design, especially when dealing with heterogeneous PPE waste.

## 5. Recycling Pathways for PPE Polymers

When considering the recycling of disposable PPE, the choice of method depends heavily on several factors encountered at each stage of the process. The properties of the material recovered, whether polypropylene masks, PET or polycarbonate face shields, or multilayered gowns, are influenced by the device’s prior use, potential contamination, and any sterilization it has undergone. Equally important are the upstream steps: collection, sorting, and pretreatment (such as cleaning, shredding, or decontamination). In this context, an additional and often critical challenge arises from the heterogeneous polymer composition of PPE, as many devices combine multiple polymers with limited thermodynamic compatibility, which directly affects the feasibility and performance of recycling processes. To clarify how polymer–polymer incompatibility constrains the recycling of multi-material PPE, Table 3 summarizes the typical compatibility relationships among the main polymers used in protective equipment and their implications for recycling outcomes.

It should be noted that the compatibility trends reported in Table 3 primarily refer to mechanical recycling pathways, where polymer immiscibility and interfacial adhesion critically determine recyclate performance. In contrast, chemical recycling routes such as dissolution, solvolysis, or pyrolysis are governed by different constraints and are less affected by polymer–polymer incompatibility. The relevance of polymers compatibility is shown in Table 4.

**Scheme 2:**
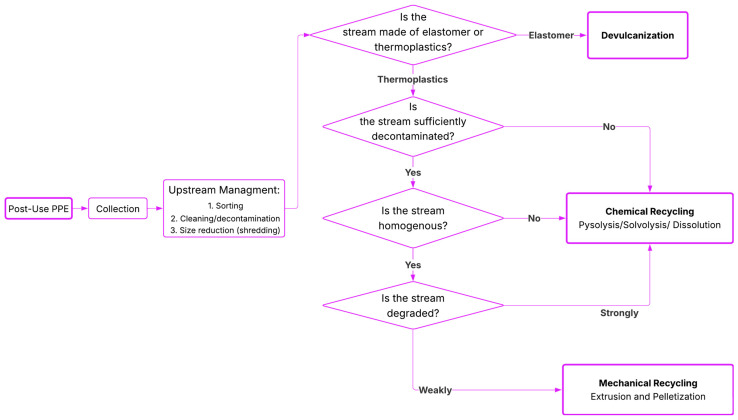
Decision-making framework for the selection of recycling routes for post-use PPE waste.

As shown in Scheme 2, depending on these variables, different recycling pathways become feasible. At one end of the spectrum, mechanical recycling can reprocess relatively clean, homogeneous polymers through shredding, extrusion, and pelletization, typically producing materials suitable for non-critical applications. Chemical recycling approaches such as pyrolysis, solvolysis, or dissolution–precipitation can recover monomers or high-quality polymers from complex or contaminated PPE waste when thermal, chemical, or mechanical degradation has occurred, or when higher-purity output is needed. Each option carries specific requirements, limitations, and potential end uses, making it essential to match the recycling strategy to the actual material condition and the desired quality of the recycled product.

### 5.1. Mechanical Recycling

Since the onset of the COVID-19 pandemic, the world has seen an unprecedented demand for disposable PPE, especially single-use masks. This surge has generated a massive quantity of plastic waste, mostly polypropylene (PP), a polymer with high market value but historically low recycling rates [88].

Yet this widespread generation of mask waste also reveals an opportunity: because many masks are made from relatively “pure” PP (nonwoven, without complex additives) and are used only briefly, they could, under the right conditions, be reclaimed and reused as a secondary raw material.

Mechanical recycling is the process of physically recovering and reprocessing plastic waste without altering its chemical structure. This typically involves cleaning, shredding, and melting the waste, followed by pelletizing to produce reusable plastic for new products.

Many studies show that recycled plastics can be used in a mixed group of plastics, and their strength and heat properties have been evaluated for specific uses [105,106,107]. The cost of recycled polymers is typically lower than that of virgin polymers; according to Torkelis et al. [108], the price gap varies widely depending on polymer type, recyclate quality, and market conditions, with a price reduction of 70% for PP and 40% for PS. This makes them an economically and environmentally sound choice, as it reduces our reliance on non-renewable resources. Nevertheless, recycled polymers frequently demonstrate substandard mechanical properties in comparison to virgin materials, a consequence of degradation during processing. These effects are mitigated by strategies such as fillers being added or blends being produced with virgin polymers [109].

One way to reduce the impact of the recycled polymer’s properties on the final manufactured product is to add fillers or produce blends [110,111]. PP is one of the most commonly sold types of plastic. The low cost, recyclability, and high thermal stability of the product make it an advantage, as it can be used to produce various blends. However, like other polymeric materials, the mechanical and thermal properties of PP are degraded by the recycling process due to the high temperatures and shear involved [112]. Recent studies on the properties of polymer blends based on virgin (vPP) and recycled PP (rPP) demonstrate variations in mechanical properties depending on the proportions used. Infurna et al. [88] investigated the mechanical recycling of polypropylene from 3-ply disposable masks, including sanitization, shredding, extrusion, and blending with virgin PP. They found that blending with virgin PP can partially restore properties, though results depend on composition. The masks’ multilayer structure was not separated, simplifying processing but increasing heterogeneity. Overall, the study showed mechanical recycling is feasible for non-critical applications, but challenges remain regarding degradation, material variability, and limited performance compared to virgin PP.

Several authors have examined the influence of virgin and recycled polypropylene (vPP/rPP) ratios on the mechanical and thermal performance of the resulting blends. Gabriel and Tiana [113] demonstrated that a formulation containing 30 wt.% rPP and 70 wt.% vPP yielded optimal results across four evaluated mechanical properties. Conversely, Hyie et al. [114] reported that a blend composed of 25 wt.% rPP and 75 wt.% vPP provided the most favorable balance, leading to enhanced tensile strength, elongation at break, and Young’s modulus. Stoian et al. [115] observed an approximate 20% increase in tensile strength, together with an improvement in elastic modulus, for blends containing 50 wt.% vPP. Moreover, all investigated compositions exhibited improved thermal stability relative comparative to neat recycled polypropylene. These studies highlight that a careful optimization of vPP/rPP blend ratios can significantly improve interfacial compatibility, mechanical performance, and thermal resistance of polypropylene-based systems.

### 5.2. Chemical Recycling

**Figure 1:**
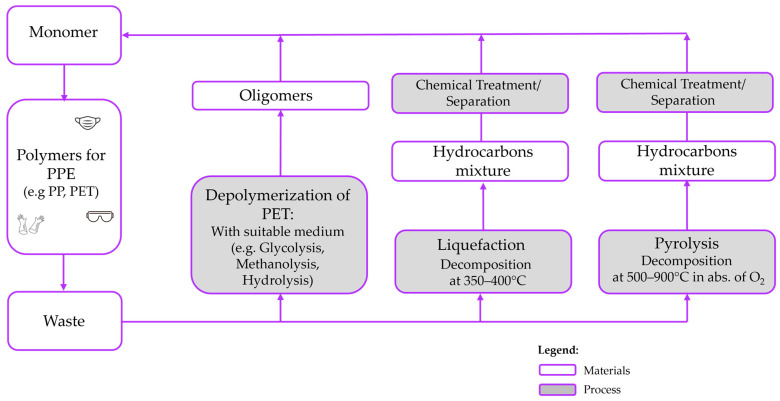
Process for chemical recycling of PPE.

In chemical recycling, long-chain polymers are broken down into valuable monomers or chemical feedstocks. This process does not require contaminated materials to be sorted beforehand, and, unlike mechanical recycling, there is no issue of having a degraded material resulting from the process This method is especially promising for PPE waste, which frequently consists of numerous polymer layers with pollutants that exceed the restrictions of traditional mechanical recycling. The main schematization of chemical recycling is shown in Figure 1.

Pyrolysis is a form of chemical recycling in which a material is thermally decomposed in the absence, or near absence, of oxygen. In the absence of oxygen, the material decomposes to produce gases (syngas), liquids (oils and tars), and solid residues (char). This process is widely used in waste treatment, biochar production, plastic recycling, and converting biomass into fuel [116,117].

The pyrolysis thermal decomposition of mask waste (largely polypropylene) at elevated temperatures in the absence of oxygen yields a spectrum of valuable products suitable for material and energy recovery [118,119]. The primary pyrolytic oil produced is suitable for repolymerization into virgin-grade polypropylene or polyethylene, offering a direct pathway to circular production of commodity polymers [120,121]. The composition of pyrolysis oil obtained from polypropylene feedstock differs substantially from conventional naphtha due to its high olefinic content, featuring cyclic olefins with bromine numbers of 85 to 304, compared to the paraffinic composition of light naphtha [122]. Although this oil is not suitable for use as a feedstock in commercial naphtha crackers for producing olefins, it can still provide valuable insights into evaluating chemical recycling pathways and determining the compatibility of recycled liquids with existing steam-cracking feedstocks. Beyond liquid products, the pyrolysis process generates valuable gas fractions usable directly as process energy, improving overall process economics and sustainability. The solid char residue remaining after pyrolysis can be valorized as a carbon filler for composite materials or as a fuel precursor [123]. For masks that are too contaminated for mechanical recycling, such as multilayer face masks with embedded adhesives, elastomers, or biohazard residues, pyrolysis is a particularly robust solution. It achieves liquid product yields of around 75% and char yields of approximately 10% at optimized reaction temperatures [123]. The high heating value of the resulting pyrolysis oil, approaching 2545 MJ/kg [124], makes it valuable as an alternative fuel for various industrial applications, including power generation and transportation fuel blending.

**Figure 2:**
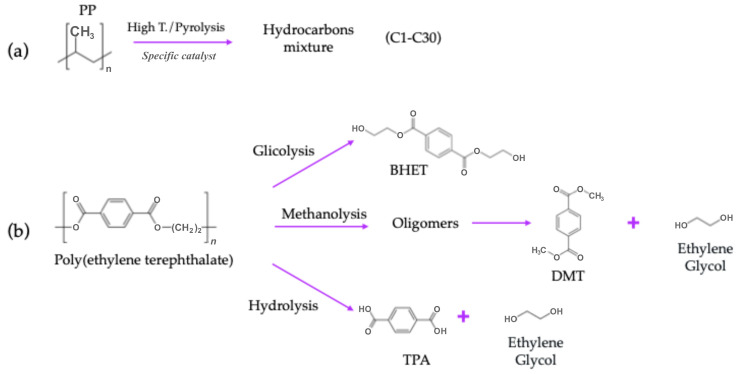
Depolymerization reaction pathway for (**a**) polyolefin (e.g., PP, PE, etc.) and (**b**) polyester (e.g., PET).

Solvolysis is defined as a chemical process in which a polymer is broken down through a reaction with a solvent that actively participates in breaking the polymer’s chemical bonds [125]. This makes it particularly relevant within the broader field of chemical recycling, where the objective is not merely to remelt and reshape plastics, but rather to revert them to their fundamental chemical components. The depolymerization of certain plastics into monomers or other valuable intermediates can be achieved through the use of specific solvents under controlled conditions. These intermediates can then undergo a process of purification, after which they can be utilized again in the production of materials that possess properties equivalent to those of virgin polymers. Consequently, solvolysis provides a method for achieving more circular and high-quality recycling, particularly for polymers that are not effectively processed by mechanical methods. For PP and PET, a reaction pathway is shown in Figure 2. This chemical depolymerization pathway proves particularly suitable for polyester-based PPE components, specifically PET and PETG face shields and visors. For low-quality PET components, methanolysis is particularly suited, while hydrolysis yields terephthalic acid and ethylene glycol, the same monomers used to produce new PET [126]. Understanding depolymerization kinetics is essential for developing commercially viable processes. Recent work has introduced a bio-solvolysis process that uses bio-based monoethylene glycol as the depolymerizing solvent, enabling a more sustainable approach to the recycling of complex PET waste containing fillers and colorants. The reaction showed high PET conversion (over 91%) and substantial bis(2-hydroxyethyl) terephthalate (BHET) monomer yields (up to 76%), followed by an activated-carbon purification step that dramatically improved color and reduced metal contaminants. Analytical characterization confirmed the high purity of the recovered monomers, with BHET reaching 99.5 mol%, demonstrating the potential of this approach to generate high-quality feedstock for closed-loop PET recycling [127]. Hyo Won Lee et al. [128] demonstrated a sustainable approach to chemically recycling PC using simple amines, without the need for solvents or catalysts. The process efficiently depolymerized BPA-based PC, including post-consumer products with additives or contaminants, yielding high amounts of bisphenol A (BPA) and urea derivatives. Both thermal and mechanochemical (ball-milling) methods were used and were effective, highlighting the versatility of aminolysis. The approach offers a green, economically attractive route to recover valuable monomers, while avoiding hazardous reagents with limitations relative to the type of amine used and the need for further scale-up studies for industrial application.

Another way to chemically recycle plastic waste is via dissolution–reprecipitation methods, which represent a gentler alternative to full chemical depolymerization, enabling selective recovery of purified polymers from multilayer PPE without extensive chain scission [129,130,131]. The solvent-targeted recovery and precipitation (STRAP) process exploits differential polymer solubility in carefully selected solvent systems to sequentially dissolve and separate the constituent layers of multilayer films [129]. By employing a computational framework integrating molecular-scale models with process modeling and life cycle assessment, the STRAP process can be optimized for complex multilayer structures containing polypropylene, polyethylene, ethylene vinyl acetate copolymer, and polyamide layers. Recent studies also use “smart” solvents (Switchable Hydrophilicity Solvents—SHSs) for the delamination of flexible multilayers with excellent recovery efficiency of separated plastic films [132]. This offers a recoverable, environmentally friendly delamination approach for multilayer flexible packaging waste, with N,N-dimethylcyclohexylamine demonstrating particular effectiveness in recovering polypropylene, polyethylene, and polyethylene terephthalate layers with high purity and minimal additive contamination. Dissolution–reprecipitation methods are patented technologies and processes with the potential to be adapted for the recycling of plastics from PPE, in consideration of the challenges outlined above. However, there is currently a notable absence of published evidence that would substantiate the utilization of these technologies on post-use PPE in a systematic or real-world manner. These gentler dissolution–reprecipitation approaches preserve the mechanical properties and chemical integrity of recovered polymers more effectively than pyrolysis, enabling multiple recycling cycles and facilitating the production of high-quality recycled materials from complex multilayer PPE waste streams [133].

Each recycling pathway addresses specific material challenges encountered in PPE waste. Pyrolysis suits contaminated or mixed-polymer streams where presorting is impractical. Solvolysis delivers virgin-grade monomers for direct polymer re-synthesis, achieving true circular-economy pathways. Dissolution–reprecipitation methods preserve polymer quality for applications demanding high mechanical performance while minimizing processing energy. Integration of multiple pathways according to PPE waste composition and contamination level represents the most pragmatic route to comprehensive PPE waste valorization.

#### Elastomer Recovery

A large part of the PPE waste stream is represented by nitrile gloves, which require another type of recycling compared with PPE based on PP, HDPE, PC, or PET. Elastomer-based PPE, including nitrile gloves, natural-latex gloves, neoprene protective items, and thermoplastic elastomer (TPE) components, represents a large and heterogeneous waste stream characterized by extensive crosslinking, additives, and fillers that complicate recycling through conventional mechanical routes [26]. Chemical recycling processes therefore focus on selectively breaking or altering the crosslinked networks while preserving as much polymer integrity as possible, enabling the formation of reusable elastomer fractions with properties comparable to virgin materials.

Devulcanization, using chemical reagents, thermo-mechanical shear, or ultrasonic energy, selectively cleaves the sulfur crosslinks in NBR without degrading the polymer backbone, producing high-quality reclaimed elastomer powders suitable for secondary manufacturing. Nitrile gloves are composed of sulfur-vulcanized NBR, where the main challenge lies in cleaving sulfur crosslinks without degrading the butadiene–acrylonitrile backbone. Chemical devulcanization employs reducing agents, thiols, disulfides, or ionic liquids to attack S–S and C–S bonds, yielding reclaim with high sol and gel fractions suitable for remanufacturing [134]. Thermo-mechanical devulcanization, performed through controlled shear and heat, breaks crosslinks via mechanochemical scission while minimizing backbone rupture. Ultrasonic devulcanization introduces cavitation-induced stresses that promote selective crosslink cleavage with very low processing temperatures [135]. Devulcanization of NBR waste from disposable gloves via chemo-mechanical methods enables breakage of sulfur crosslinks while retaining the polymer backbone, producing reclaimed elastomer with improved mechanical properties suitable for reuse in rubber products such as mats, composites, and molded goods [26]. Studies on blends of devulcanized NBR via sulfur/peroxide mixture from glove waste with virgin rubber indicate that the inclusion of reclaimed NBR affects cure characteristics and mechanical performance, offering a sustainable pathway for repurposing PPE elastomeric waste [26]. Recycling strategies applied to natural rubber latex waste demonstrate that devulcanized rubber can retain crosslink density and mechanical integrity after reprocessing, suggesting that similar approaches could be applied to latex PPE waste [136]. Latex gloves can be recycled through chemical peptization, which breaks down the gel structure of vulcanized natural rubber and restores its processability. The recovered nitrile and natural rubber fractions can be reintegrated into value-added applications such as rubber composites, footwear components, anti-fatigue mats, road-pavement modifiers, and molded industrial products, offering a circular strategy for managing elastomeric PPE waste while reducing the need for virgin rubber [137].

## 6. Upcycling and Downcycling Opportunities

**Figure 3:**
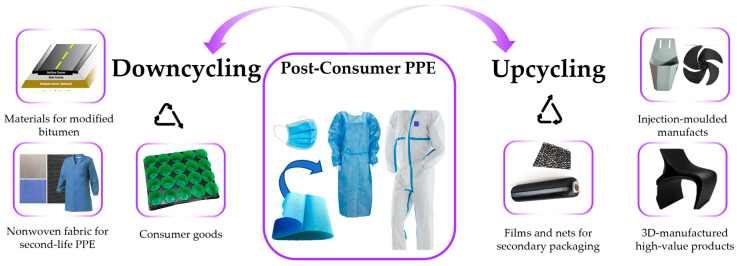
Potential applications for post-consumer PPE.

In the context of post-consumer PPE, material recovery can follow different pathways with distinct implications for functionality and value, as is shown in Figure 3. According to principles aligned with ISO 14021:2016 on environmental claims and the EU Circular Economy framework [138], upcycling refers to recycling processes that maintain or enhance the functional, technical, or economic value of the recovered material relative to its original application. Upcycling strategies, which may include mechanical, chemical, or hybrid routes, allow PPE polymers and fibers to be reintroduced into high-value applications, preserving their protective or mechanical properties and contributing to resource efficiency and circularity. In contrast, downcycling describes pathways in which the recovered materials are transformed into products with reduced performance, quality, or value, often due to contamination, material heterogeneity, or degradation during use and reprocessing. Downcycling, while still enabling material recovery, generally limits secondary applications to non-critical or low-performance uses, representing a lower level of value retention within the waste hierarchy.

From a materials perspective, the distinction between upcycling and downcycling is closely related to the performance gap between a virgin material and a recycled polymer. Mechanical recycling generally induces chain scission and thermo-oxidative degradation, resulting in reduced molecular weight, broader property distributions, and increased heterogeneity. Post-consumer recycled PP exhibits increased MFI and modified mechanical performance compared to virgin PP. Zhiltsova et al. [139] found that MFI values of 100% recycled PP were significantly higher than virgin PP, indicating lower molecular weight and changes in rheological behavior due to recycling [88]. Recycling generally results in diminished tensile strength and impact resistance for recycled PP relative to virgin grades, although rigidity may increase in some cases [140]. Studies on blends of virgin and recycled PP demonstrate that low-to-moderate recycled content can achieve mechanical performance close to virgin materials, while higher recycled content tends to amplify performance loss [139].

These changes often limit the direct reuse of recycled PP in high-performance nonwoven applications, favoring its redirection towards lower-demand uses or blending strategies (downcycling), whereas chemical recycling routes may partially restore virgin-like properties and enable higher-value applications (upcycling).

These conceptual distinctions between upcycling and downcycling provide the basis for interpreting quantitative environmental outcomes reported in LCA studies of PPE recycling. In particular, LCA studies on mask reprocessing demonstrate that extending the functional life of PPE through sterilization and reuse can lower the carbon footprint by approximately 50–60% per functional unit, despite the additional energy demand associated with decontamination steps, which typically represents a minor share (<20%) of total life cycle impacts [141,142]. At the system level, analyses of healthcare PPE supply chains indicate that material recycling scenarios (mechanical recycling of polypropylene components) can reduce greenhouse gas emissions by ~30–35% relative to baseline disposal via high-temperature incineration, while combined strategies including reuse, recycling, and supply-chain optimization may reach reductions of up to 70–75% [143]. From an energy perspective, mechanical recycling of PPE plastics typically requires one order of magnitude less cumulative energy demand than virgin polymer production, whereas chemical recycling routes show intermediate performance, with higher energy inputs but improved material quality. These differences are reflected in the distinction between downcycling, where recycled PPE is converted into lower-value plastic products with moderate environmental gains, and upcycling, where higher-quality recyclates or blends partially substitute virgin polymers, leading to substantially greater climate and resource benefits per unit of material recovered.

The upcycling approach can be implemented through different processing routes, encompassing mechanical recycling methods (such as size reduction followed by melt reprocessing), chemical modification techniques (including devulcanization and surface or bulk functionalization), and thermochemical pathways (notably pyrolysis and carbonization). These processes are often combined with sanitization and decontamination steps to address biological risks, depending on the waste’s origin [144]. The PP fiber from the outer layers of masks can be shredded and recomposed as a matrix or reinforcement in thermoplastic/thermosetting composites. It has been demonstrated through experimental studies that, when subjected to appropriate compatibilization treatments, rPP from masks can exhibit favorable mechanical properties, enhance impact resistance, and facilitate the utilization of lightweight structural applications, ranging from the reinforcement of glass fiber composites to sheet molding and three-dimensional printing [27,145]. Following the separation process, the meltblown layer of masks (PP microfibers), which constitutes the inner layer, can be isolated and reused in fibrous panels for filtration or acoustic insulation applications. An initial comparative investigation was carried out to evaluate the acoustic absorption performance of surgical face masks in relation to conventional fibrous materials commonly used in building applications [146]. The findings indicated that this fibrous waste could potentially serve as a substitute for traditional materials, demonstrating the fibrous waste’s favorable acoustic performance and its potential as a substitute for conventional fibrous materials in construction and soundproofing applications.

In the existing literature, an alternative method for the upcycling of PP-based PPE has been documented. This method involves the carbonization of polypropylene face masks in the presence of sulfuric acid, high-quality submicron-sized graphite powder [147], or carbon-based thin films [148]. The process is accomplished through the utilization of carbonization in the presence of sulfuric acid. In the case of submicron-sized particles, the process is further augmented by the incorporation of high-energy ball milling. This approach enables the synthesis of carbon-based materials with elevated added value, offering a sustainable and economically viable pathway for the valorization of polypropylene waste in supercapacitor applications.

Devulcanized rubber has been identified as a potential constituent in the composition of acrylonitrile butadiene styrene copolymer (ABS) and thermoplastic polyolefins (TPO), with the objective of enhancing the functionality of the resultant materials through the utilization of 3D printing techniques. The incorporation of devulcanized rubber has been shown to yield thermoplastic polyolefin-based composites that exhibit high-impact properties, with an elongation at break exceeding 500% and commendable compression properties, accompanied by an excellent shape recovery ratio post-compression [149]. Upcycling reduces landfill/incineration volume and produces commercially valuable products (construction panels, 3D filaments, electrodes, acoustic materials). Preliminary cost–benefit analyses suggest economic potential if safe collection and operational scales are implemented [27].

In the context of contaminated or highly mixed PPE waste streams (e.g., masks, gowns, gloves), downcycling remains an essential strategy because purification or high-quality recycling is often impractical due to contamination, polymer degradation, and heterogeneous composition. Though downcycling does not achieve closed-loop recycling, it contributes to diverting large volumes of PPE from landfills and incineration, reducing environmental impact. For PPE streams that cannot be effectively cleaned or sorted for high-value recycling, repurposing into construction and other low-grade applications can represent a realistic end-of-life pathway; although this approach does not enable closed-loop recycling, it can still play a role within a broader circular-economy framework as an open-loop or cascading strategy aimed at extending material use and reducing landfill disposal. Waste PPE, especially PP from masks and gowns, can be shredded and incorporated into cementitious composites or mortar to serve as reinforcement fibers [150]. For instance, recycled polypropylene fibers extracted from surgical face masks have been successfully used to improve the tensile and flexural strength of cement mortar mixes, demonstrating that shredded mask fibers can enhance the mechanical performance of construction materials [151]. Further studies on shredded waste mask fibers have shown modified mechanical properties, including toughness and deformation behavior, highlighting their potential use in non-structural or engineered cementitious materials [152]. These fibers have also been used in polymer-modified bitumen compatibilized with maleic anhydride, significantly enhancing its performance and indicating that polypropylene derived from masks is a technically viable and sustainable alternative to virgin polypropylene in the production of polymer-modified bitumen, thereby improving binder performance [153]. When PPE cannot meet quality standards for structural materials, it can be melt-processed or blended with virgin plastics to manufacture low-grade consumer products. These include garden waste bins, utility mats, and non-load-bearing handles. While the mechanical properties are typically inferior to those of virgin polymers, such reuse displaces new plastic production and extends waste utility. The concept of incorporating mixed or contaminated plastic waste into durable goods aligns with broader recycling practices [88].

Reclaimed polypropylene derived from single-use PPE represents a promising secondary raw material capable of partially substituting virgin polymers in a wide range of applications [154]. Its valorization is primarily driven by the combined benefits of material efficiency, energy reduction, and environmental mitigation. From a resource management perspective, the recovery of polymeric fractions from discarded face masks contributes to lowering the consumption of fossil-based feedstocks and reduces reliance on energy-intensive virgin plastic production routes [155,156]. In addition, mechanical recycling pathways for mask-derived plastics exhibit substantially lower energy requirements when compared to primary polymer synthesis, resulting in improved process efficiency and reduced embodied energy of the final materials [157]. Beyond resource and energy considerations, the reuse of waste mask plastics offers relevant environmental co-benefits, including the mitigation of emissions and pollutant release associated with raw material extraction and processing. Furthermore, diverting PPE waste from uncontrolled disposal routes may limit microplastic generation and potential human and ecosystem exposure [158]. Overall, the recycling and reintegration of PPE-derived polymers is consistent with circular-economy strategies, promoting material retention within the value chain while minimizing waste and preserving resources [159].

## 7. Conclusions

The evidence clearly shows that the large and continuous flow of PPE waste, driven by ongoing healthcare needs and high-tech industrial demands, is one of the most significant challenges to the transition to a circular economy. The conventional linear model of “take, make, use, dispose” is financially and ecologically unsustainable, particularly when dealing with large volumes of polymer-rich waste, such as masks, gloves, and gowns, which often default to costly incineration due to contamination concerns. The primary barrier to achieving circularity is the heterogeneity of PPE materials (PP, NBR, PC, etc.) and, crucially, the sector-specific contamination profiles (biohazardous waste in healthcare, chemical/solvent residues in laboratories, particulate matter in cleanrooms). This inherent complexity frequently results in the default disposal method being costly and energy-intensive incineration, particularly for high-risk streams. The efficacy of recycling is contingent upon the implementation of effective source segregation. The implementation of pilot programs has demonstrated that the segregation of “non-infectious” PPE can result in the generation of clean, economically viable feedstocks. However, it is imperative to exercise caution when employing decontamination processes, such as autoclaving or chemical washing, as these must be meticulously aligned with the polymer type. Inadequate treatment can result in the degradation of material integrity, consequently rendering it unsuitable for high-quality reprocessing. The streams from food processing and cleanrooms are characterized by enhanced cleanliness and compositional uniformity (predominantly polyolefins such as PE/PP), rendering them optimal candidates for immediate mechanical recycling via closed-loop or semi-closed-loop systems. In contrast, the streams from healthcare and laboratory settings are classified as high-risk, necessitating the implementation of advanced pathways. The economic pressure from high disposal costs (which can reach up to 10 times higher than those of landfilling) provides a strong financial incentive for the development of robust segregation and advanced treatment solutions. A number of recycling methods were discussed in the context of the nature of the waste stream. It was concluded that mechanical recycling is more effective for clean, homogeneous streams, such as PP masks and HDPE suits from cleanrooms. In contrast, contaminated or mixed-polymer streams, such as multilayer masks and PC face shields, are better suited to chemical recycling. Furthermore, a discourse on the subject of elastomerics illuminates the potential for selective devulcanization of nitrile or latex gloves, a process that involves the deliberate rupture of crosslinks. This method yields a high-quality reclaimed elastomer powder, which can be utilized in industrial composites and molded goods. In relation to the nature of the streams and the type of recycling employed, the following processes can be performed: upcycling, whereby PPE-derived fibers are transformed into high-value components (e.g., acoustic insulation, 3D printing filaments, carbon-based supercapacitors) and downcycling, whereby PPE-waste shredded fibers are incorporated into construction materials (e.g., cementitious composites, bitumen modifiers). These processes divert large volumes of waste away from incineration. The strategic adoption of multi-pathway approaches has the potential to transform the disposal challenge posed by PPE waste into a significant, sustainable source of secondary raw materials. This, in turn, would contribute directly to resource conservation and the realization of the EU’s Green Deal objectives.

Furthermore, the strategies outlined in this work align directly with several United Nations Sustainable Development Goals (SDGs). Circular management of PPE waste contributes to SDG 12: Responsible Consumption and Production, by promoting resource efficiency and reducing landfill and incineration. It also supports SDG 13: Climate Action, through the mitigation of greenhouse gas emissions associated with conventional disposal, and SDG 9: Industry, Innovation, and Infrastructure, by fostering advanced recycling technologies and industrial valorization of secondary raw materials. By framing PPE circularity within the SDG framework, this work highlights its potential not only for local or sectoral sustainability but also for global environmental and socio-economic impact.

In order to unlock the full potential of circular PPE, regulatory frameworks must evolve to recognize safe recycled materials, and industries must adopt design-for-recycling principles. It is concluded that with these changes, PPE, once emblematic of single-use culture, could become a showcase example of how complex polymer products can be reintegrated into sustainable material loops.

## Figures and Tables

**Table 1:** Comparison of sorting technologies for mixed post-consumer PPE waste.

	Sorting Accuracy	Throughput	Cost	Note	Refs.
NIR Spectroscopy	High for homogenous plastics (PE, PP, PET): >90%	Up to 10 t/h	Moderate to high; initial investment EUR 200k–EUR 500k per line; low operational cost.	Commonly used for films, gloves, and aprons; limitations with multilayer PPE or composites.	[75]
Raman Spectroscopy	Very high: >95%	0.5–2 t/h	High: equipment cost EUR 300k–EUR 700k; requires specialized operator.	Excellent for complex PPE, e.g., multilayer masks, laminated aprons. Sensitive to surface contamination.	[76,77]
Tribo-Electrostatic Separation	Moderate: ~80–85%	Up to 5–6 t/h	Moderate: capital cost EUR 100k–EUR 200k; low operating costs, but pretreatment may be needed (drying, shredding).	Best for separating polymers with different tribo-electric properties; less effective for mixed contamination or small fragments.	[78,79]
Manual Sorting/Visual	Low to moderate	0.5–1 t/h	Low initial cost, high labor cost.	Often used as presorting; insufficient alone for high-value recycling.	

**Table 2:** Comparison of mechanical and chemical pretreatment methods for PPE waste recycling.

Pretreatment	Descriptions	Advantages	Limitations	Applicability
Mechanical	Shredding, washing, drying, densification/agglomeration	- Increases surface area - Homogenizes feedstock - Removes surface residues - Facilitates extrusion	- Can introduce structural defects or microplastics - Incomplete homogenization reduces mechanical properties - Sensitive materials may degrade	All PPE types (films, nonwovens, gloves, rigid plastics)
Chemical	Solvent washing + selective dissolution/precipitation. Removes contaminants and additives, recovering a clean polymer	- Produces high-purity recyclate - Preserves polymer integrity - Suitable for mixed and composite waste streams	- Requires preliminary sorting - Additives and non-polymer components may remain - Solvent management needed (costs, safety, sustainability)	Contaminated PPE, multilayer films, composite materials, mixed plastic streams

**Table 3:** Polymer compatibility and incompatibility mechanisms in PPE recycling.

	Typical Miscibility	Main Incompatibility Mechanisms	Implications for PPE Recycling	Ref.
PP/PE	Low	Phase separation, weak interfacial adhesion	Moderate property loss in mixed streams	[101]
PP/PET	Very low	Strong phase separation, brittleness	Severe downcycling without compatibilization	[102]
PE/PET	Very low	Immiscibility, poor stress transfer	Limited recyclability in mixed PPE waste	[103]
PP/PC	Very low	Polarity mismatch, viscosity mismatch	Severe embrittlement, phase-separated morphology	[101]
PE/PC	Very low	Immiscibility, poor adhesion	Mechanical property collapse	[101]
PET/PC	partially miscible	Phase separation at certain compositions	Potential for upcycling with compatibilization	[104]

**Table 4:** Relevance of polymer compatibility across different PPE recycling routes.

Recycling Route	Role of Polymer Compatibility	Implications for Mixed PPE Waste
Mechanical recycling	Critical	Immiscibility causes phase separation, embrittlement, and downcycling
Dissolution-based recycling	Limited	Selective polymer recovery possible; multilayer structures manageable
Solvolysis	Low	Polymer-specific reactions dominate over blend compatibility
Pyrolysis	Negligible	Mixed polymers can be processed, but product quality is downgraded to fuels/chemicals

## Data Availability

No new data were created or analyzed in this study. Data sharing is not applicable to this article.
